# Undernutrition and associated factors among pregnant women attending antenatal care at Awash Sebat Health Center, Afar Region, Ethiopia: A facility-based mixed-methods study

**DOI:** 10.1097/MD.0000000000050636

**Published:** 2026-09-11

**Authors:** Temesgen Gebeyehu Wondmeneh, Molla Kahssay

**Affiliations:** a Department of Public Health, College of Medical and Health Science, Samara University, Semera, Ethiopia.

**Keywords:** Afar, associated factors, Ethiopia, pregnant women, undernutrition

## Abstract

Maternal undernutrition remains a major public health problem in low- and middle-income countries, including Ethiopia, where it contributes substantially to adverse maternal and neonatal outcomes. This study aimed to determine the prevalence of undernutrition, identify associated factors, and explore cultural food taboos among pregnant women attending antenatal care (ANC) at Awash Sebat Health Center, Afar Region, Ethiopia. A facility-based convergent mixed-methods study was conducted among pregnant women attending ANC. Systematic random sampling was used to recruit participants for the quantitative component, while purposive sampling was employed for focus group discussions. Descriptive statistics were used to summarize participant characteristics. Bivariable and multivariable logistic regression analyses were performed to identify factors associated with maternal undernutrition. Qualitative data were analyzed using the Framework method. The prevalence of maternal undernutrition was 38.1%. Maternal undernutrition was associated with higher odds among illiterate women (adjusted odds ratio [AOR] = 4.20, 95% confidence interval [CI]: 1.50–10.80), those with a monthly household income below 1500 Ethiopian birr (AOR = 5.62, 95% CI: 2.40–15.98), a birth interval of less than 2 years (AOR = 3.70, 95% CI: 1.15–12.50), no prior ANC utilization (AOR = 5.88, 95% CI: 1.35–25.00), and low dietary diversity (AOR = 3.45, 95% CI: 1.78–6.89). In contrast, women from households with 4 or fewer members had significantly lower odds of undernutrition (AOR = 0.17, 95% CI: 0.08–0.71). Qualitative findings indicated that many pregnant women avoided protein-rich foods, fruits, vegetables, milk products, and semi-solid foods because of cultural beliefs that these foods increase fetal size, complicate childbirth, or adhere to the fetus. These beliefs appeared to contribute to poor dietary diversity, although food taboos were not independently associated with undernutrition. More than one-third of pregnant women were undernourished. Maternal education, household income, family size, birth spacing, ANC utilization, and dietary diversity were independently associated with maternal undernutrition. These findings highlight the importance of strengthening maternal nutrition education, improving socioeconomic conditions, promoting adequate birth spacing, encouraging ANC utilization, and addressing harmful food taboos through culturally appropriate nutrition education to support maternal nutritional well-being in pastoral communities.

## 1. Introduction

Undernutrition during pregnancy is a major public health problem characterized by inadequate maternal nutrient reserves and insufficient intake of macronutrients and micronutrients required to support optimal maternal, fetal, and neonatal health. Maternal undernutrition increases the risk of adverse pregnancy and birth outcomes, including spontaneous abortion, intrauterine growth restriction, preterm birth, low birth weight, and perinatal mortality.^[1–5]^ In addition to its immediate consequences, maternal undernutrition contributes to poor maternal health, impaired fetal growth, and intergenerational cycles of malnutrition.^[1,5]^ Undernutrition and overnutrition represent 2 forms of the global burden of malnutrition and are indicators of social and economic inequalities, disproportionately affecting low- and middle-income countries.^[6]^ Maternal and child undernutrition, together with micronutrient deficiencies, continues to impose a substantial global health burden, particularly in Africa and Asia.^[7]^ In India, for example, nearly 48% of pregnant women have been reported to be undernourished.^[8]^ In Ethiopia, improving maternal nutrition remains a public health priority. Despite considerable efforts to improve maternal health, maternal undernutrition remains common in many parts of the country.^[9]^ According to the 2016 Ethiopia Demographic and Health Survey, 22.4% of women of reproductive age were undernourished nationally, while the Afar Region reported the highest prevalence (39.1%).^[10]^ Similarly, facility- and community-based studies conducted in different parts of Ethiopia reported undernutrition prevalences ranging from 16.2% in Gondar,^[11]^ 19.1% in eastern Ethiopia,^[12]^ and 28.6% in the Gambella Region.^[13]^ These findings indicate substantial geographical variation in maternal nutritional status across the country.

Previous studies have identified several determinants of maternal undernutrition, including low educational attainment, poor household income, larger family size, short birth intervals, inadequate dietary diversity, limited meal frequency, and inadequate antenatal care (ANC) utilization.^[8,14–21]^ In addition to these socioeconomic and reproductive factors, cultural beliefs and food taboos during pregnancy may restrict the consumption of nutritious foods, thereby reducing dietary diversity and potentially compromising maternal nutritional status.^[14]^ However, these cultural influences have received limited attention in previous quantitative studies.

The Afar Region is characterized by recurrent drought, food insecurity, a predominantly pastoral lifestyle, and unique sociocultural practices that may influence maternal nutrition. Although regional estimates of undernutrition are available from the Ethiopia Demographic and Health Survey, evidence regarding the factors associated with maternal undernutrition and the influence of cultural food taboos among pregnant women attending ANC in this setting remains limited. A mixed-methods approach provides an opportunity to quantify the burden of undernutrition and its associated factors while simultaneously exploring the cultural beliefs and food practices that may influence maternal dietary behaviors. Integrating quantitative and qualitative findings can therefore provide a more comprehensive understanding of maternal undernutrition in this pastoral community.

Therefore, this study aimed to determine the prevalence of undernutrition, identify its associated factors, and explore pregnancy-related food taboos among pregnant women attending ANC at Awash Sebat Health Center, Afar Region, Ethiopia, using a facility-based mixed-methods study design.

## 2. Methods

### 2.1. Study design

A facility-based convergent mixed-methods cross-sectional study was conducted to determine the prevalence of undernutrition, identify associated factors, and explore cultural food taboos among pregnant women attending ANC. The quantitative and qualitative components were conducted concurrently, analyzed separately, and integrated during interpretation to provide a comprehensive understanding of maternal undernutrition in the study setting.

### 2.2. Study setting

The study was conducted at Awash Sebat Health Center, Afar Region, northeastern Ethiopia, from September 1, 2019, to November 5, 2019. The Afar people are one of the indigenous pastoral communities in the Horn of Africa and predominantly speak the Afar language. Most residents rely on pastoralism for their livelihood, while crop production is limited because of the arid and semiarid climatic conditions. Recurrent drought, extreme temperatures, and food insecurity are common challenges affecting the region. During the dry season, livestock migrate to major rivers in search of pasture and water. The Afar economy primarily depends on livestock production, particularly goats, camels, and cattle.^[22,23]^ The Afar social structure is based on patrilineal clan organization, in which members share resources and provide mutual support during times of hardship.^[24]^ To improve food security among vulnerable households, the Ethiopian government and development partners have implemented the Productive Safety Net Programme–Pastoral Areas Pilot since 2009.^[25]^

### 2.3. Study population, sample size, and sampling procedure

The quantitative component included pregnant women who attended ANC services at Awash Sebat Health Center during the study period, including women attending their first ANC visit. The qualitative component included pregnant women purposively selected for focus group discussions (FGDs) based on their ability to provide rich information regarding cultural food practices during pregnancy.

The sample size for the quantitative study was determined using the single population proportion formula assuming a 95% confidence level, a 5% margin of error, and a prevalence of maternal undernutrition of 19.1% reported from eastern Ethiopia.^[12]^ After adding a 10% allowance for possible nonresponse, the final sample size was 261 pregnant women. Based on the ANC registration records, approximately 412 pregnant women attended the health center during the study period. The sampling interval (k) was calculated as approximately 2 (412/261). After selecting the first participant by lottery from the first 2 eligible women, every second eligible pregnant woman was recruited using systematic random sampling until the required sample size was achieved. Women who had already participated in the study during a previous ANC visit were not interviewed again.

For the qualitative component, purposive sampling was used to recruit pregnant women with different ages, educational backgrounds, parity, and places of residence to capture diverse experiences. Six FGDs comprising 6 participants each (n = 36) were conducted. Data collection continued until thematic saturation was achieved, with no new information emerging from subsequent discussions. Participants in the qualitative study were different from those included in the quantitative survey.

### 2.4. Eligibility criteria

All pregnant women attending ANC services at Awash Sebat Health Center who had lived in the study area for at least 6 months and provided informed consent were eligible for inclusion. Pregnant women diagnosed with chronic illnesses, including diabetes mellitus and cancer, were excluded because these conditions may independently influence dietary intake and nutritional status. Information regarding chronic illnesses was obtained from participants’ medical records and self-reports.

### 2.5. Data collection procedures

#### 2.5.1. Quantitative component

Data were collected using a structured interviewer-administered questionnaire developed after reviewing relevant literature. The questionnaire included information on sociodemographic characteristics, socioeconomic status, obstetric history, maternal healthcare utilization, dietary practices, and cultural food taboos. The independent variables included maternal age, religion, ethnicity, marital status, place of residence, family size, educational status, occupation, average monthly household income, birth interval, parity, gestational age, ANC utilization, food taboos, meal frequency, and dietary diversity. ANC utilization was categorized according to the number of ANC visits received during the current pregnancy before the interview. Women attending ANC for the first time were classified as having no prior ANC utilization, while those who had previously attended ANC were categorized as having 1 to 2 or 3 to 4 prior ANC visits. The dependent variable was maternal nutritional status, assessed using mid-upper arm circumference (MUAC). Pregnant women with MUAC <22 cm were classified as undernourished, whereas those with MUAC ≥22 cm were classified as having normal nutritional status.^[26]^ Dietary diversity was assessed using a modified version of the Food and Agriculture Organization dietary diversity questionnaire.^[27]^ The questionnaire was originally prepared in English, translated into the Afar language by a bilingual native speaker, and back-translated into English to ensure consistency. Prior to data collection, the questionnaire was pretested outside the study area, and necessary modifications were made. Three trained health professionals (2 midwives and 1 nurse) collected the quantitative data under the supervision of the principal investigator. Face-to-face interviews were conducted in a private setting to maintain confidentiality. MUAC was measured 3 consecutive times using a non-stretchable MUAC tape, and the average measurement was used for analysis to improve measurement accuracy.

#### 2.5.2. Qualitative component

The qualitative component explored cultural beliefs and food taboos related to pregnancy. Six FGDs were conducted using a semi-structured interview guide developed from the study objectives (Table 1). Each FGD included 6 pregnant women and was facilitated by trained qualitative data collectors fluent in the Afar language, while another researcher served as a note-taker. Discussions were conducted in locations convenient for participants and lasted approximately 60 to 90 minutes. All discussions were audio-recorded with participants’ permission, supplemented by detailed field notes documenting nonverbal expressions and contextual observations. Audio recordings were transcribed verbatim in the Afar language and translated into English. To improve credibility, transcripts were reviewed against the recordings for accuracy before analysis.

**Table 1:** Guiding and probing questions used during focus group discussions with pregnant women.

Participants	Main guiding question	Interview prompts (probes)
Pregnant women	Can you describe the cultural beliefs and food practices related to pregnancy in your community, including the foods that are encouraged or avoided and the reasons for these practices?	What foods are traditionally avoided during pregnancy in your community?
		Why do pregnant women avoid these foods during pregnancy?
		What cultural or religious beliefs influence food choices during pregnancy?
		What do people believe will happen if pregnant women consume these foods during pregnancy?
		What do community members or family elders advise pregnant women regarding food consumption?
		Have these food beliefs or restrictions affected your eating habits during your current or previous pregnancy? Please explain.
		What do you think could help pregnant women consume a healthy and balanced diet while respecting local traditions?

### 2.6. Operational definitions

Undernutrition: Pregnant women with a MUAC <22 cm were classified as undernourished.^[26]^

Dietary diversity: Dietary diversity was categorized as low (<4 food groups) and adequate (≥4 food groups) according to the Food and Agriculture Organization guidelines.^[27]^

### 2.7. Data analysis

Quantitative data were entered and analyzed using IBM SPSS Statistics, version 23 (IBM Corp.). Descriptive statistics, including frequencies, percentages, means, and tables, were used to summarize participants’ characteristics. Binary logistic regression analysis was initially performed to assess factors associated with maternal undernutrition. Variables with a *P*-value <.05 in the bivariable analysis were entered into the multivariable logistic regression model to identify factors independently associated with maternal undernutrition. Adjusted odds ratios (AORs) with 95% confidence intervals (CIs) were calculated, and statistical significance was declared at *P* < .05. For the logistic regression analysis, maternal nutritional status was coded as 1 for undernutrition (MUAC <22 cm) and 0 for normal nutritional status (MUAC ≥22 cm). Therefore, an AOR >1 indicates higher odds of undernutrition, whereas an AOR <1 indicates lower odds of undernutrition.

Qualitative data were analyzed using the Framework method.^[28]^ Audio recordings were transcribed verbatim in the Afar language and translated into English. Coding and thematic analysis were conducted manually using the Framework method. Two investigators independently reviewed the transcripts to achieve familiarization with the data and developed initial codes inductively. Any differences in coding were resolved through discussion and consensus. Codes were grouped into categories, from which overarching themes and subthemes were developed. Credibility was enhanced through triangulation of the qualitative and quantitative findings.

### 2.8. Ethical consideration

Ethical approval was obtained from the Ethics Committee of the Medical and Health Science College of Samara University. Permission to conduct the study was obtained from the Awash Sebat Health Center administration before data collection. Written informed consent was obtained from all participants after explaining the objectives, procedures, potential benefits, and voluntary nature of the study. Participants were informed of their right to withdraw at any stage without any consequences. Confidentiality and anonymity were maintained throughout the study by removing personal identifiers from all data collection tools. All study procedures were conducted in accordance with the principles of the Declaration of Helsinki.

## 3. Results

### 3.1. Quantitative results

#### 3.1.1. Sociodemographic characteristics of the participants

Of the 261 eligible pregnant women, 247 participated in the study, yielding a response rate of 94.6%. Approximately 29.1% of the participants were 21 to 25 years of age, and 95.1% were married. Most participants were Muslims (72.1%), while 58.7% belonged to the Afar ethnic group. Nearly 3-quarters (75.7%) resided in urban areas. Approximately 36.0% of the pregnant women were illiterate, and the majority were housewives. About 72.5% of participants lived in households with 4 or fewer family members, whereas 51.4% reported a monthly household income of 1500 to 3000 Ethiopian birr (Table 2).

**Table 2:** Sociodemographic characteristics of pregnant women.

Variables	Categories	No (%)
Age (yr)	≤20	51 (20.6)
	21–25	72 (29.1)
	26–30	69 (27.9)
	≥31	55 (22.3)
Marital status	Married	235 (95.1)
	Unmarried	12 (4.9)
Religion	Orthodox	56 (22.7)
	Protestant and Catholic	13 (5.3)
	Muslim	178 (72.1)
Ethnicity	Others	40 (16.2)
	Amhara	62 (25.1)
	Afar	145 (58.7)
Residence	Urban	187 (75.7)
	Rural	60 (24.3)
Educational status	Illiterate	89 (36)
	Primary school	70 (28.3)
	Secondary school and above	88 (35.7)
Occupation	Housewives	187 (75.5)
	Government-employed	23 (9.3)
	Merchants	37 (15)
Average monthly income (ETB)	<1500	53 (21.5)
	1500–3000	127 (51.4)
	>3000	67 (27.1)
Family size (members)	≤4	179 (72.5)
	>4	68 (27.5)

Others include Oromo, Southern Nations, Nationalities, and Peoples, and Tigray peoples.

ETB = Ethiopian birr.

#### 3.1.2. Obstetric characteristics and maternal healthcare utilization

Among multiparous women, 67.1% had a birth interval of at least 2 years. Nulliparous women accounted for 30.8% of the study participants. Approximately half of the respondents (50.2%) were in the third trimester of pregnancy (>6 months’ gestation). Women with no prior ANC visits during the current pregnancy accounted for nearly one-third (32.8%; Table 3).

**Table 3:** Obstetric and health service utilization characteristics of pregnant women.

Variables	Categories	No (%)
Birth interval	<2 yr	55 (32.9)
	≥2 yr	112 (67.1)
Parity	Nulliparous	76 (30.8)
	Multiparous	171 (69.2)
Month of pregnancy	<3 mo	38 (15.4)
	3–6 mo	85 (34.4)
	>6 mo	124 (50.2)
Antenatal care utilization	No prior antenatal care utilization	81 (32.8)
	1–2 times visits	121 (49)
	3–4 times visits	45 (18.2)

#### 3.1.3. Nutritional status and dietary practices

The prevalence of maternal undernutrition was 38.1% (94/247). About 73.7% of participants had low dietary diversity, while 57.1% had not received iron–folic acid supplementation during pregnancy. Most pregnant women reported eating 3 meals per day, and 37.7% reported practicing food taboos during pregnancy (Table 4).

**Table 4:** Nutritional status and dietary habits of pregnant women.

Variables	Categories	No (%)
Undernutrition	Yes	94 (38.1)
	No	153 (61.9)
Dietary diversity	Low	182 (73.7)
	Adequate	65 (26.3)
Taking iron or folic acid	Yes	106 (42.9)
	No	140 (57.1)
Frequency of eating per day	3 times	183 (74.1)
	4 times	42 (17)
	>4 times a day	22 (8.9)
Is there a food taboo during pregnancy?	Yes	93 (37.7)
	No	154 (62.3)

#### 3.1.4. Factors associated with maternal undernutrition

In the bivariable logistic regression analysis, maternal age, religion, ethnicity, place of residence, educational status, occupation, household income, family size, birth interval, parity, ANC utilization, frequency of eating per day, dietary diversity, and food taboo practice were significantly associated with maternal undernutrition. However, after adjusting for potential confounding variables in the multivariable logistic regression model, educational status, household income, family size, birth interval, ANC utilization, and dietary diversity were independently associated with maternal undernutrition.

Compared with pregnant women who had attained secondary school or higher education, illiterate women had significantly higher odds of being undernourished (AOR = 4.20; 95% CI: 1.50–10.80). Similarly, women with a monthly household income of less than 1500 Ethiopian birr had significantly higher odds of being undernourished than those with a monthly household income >3000 Ethiopian birr (AOR = 5.62; 95% CI: 2.40–15.98). In contrast, women living in households with 4 or fewer family members had significantly lower odds of being undernourished than those living in households with more than 4 family members (AOR = 0.17; 95% CI: 0.08–0.71). Women with a birth interval of less than 2 years had significantly higher odds of being undernourished than those with a birth interval of at least 2 years (AOR = 3.70; 95% CI: 1.15–12.50). Women with no prior ANC utilization during the current pregnancy had significantly higher odds of being undernourished than those with 3 to 4 prior ANC visits (AOR = 5.88; 95% CI: 1.35–25.00). Likewise, pregnant women with low dietary diversity had significantly higher odds of being undernourished than those with adequate dietary diversity (AOR = 3.45; 95% CI: 1.78–6.89). Although food taboo practice was significantly associated with maternal undernutrition in the bivariable analysis, the association was no longer statistically significant after adjustment for potential confounding variables (Table 5).

**Table 5:** Bivariable and multivariable logistic regression analyses of factors associated with maternal undernutrition among pregnant women.

Variables	Nutritional status			COR (95% CI)	AOR (95% CI)
	Undernourished (MUAC <22 cm)	Normal (MUAC ≥22 cm)			
	No (%)	No (%)			
Age (yr)					
≤20	16 (6.5)	35 (14.2)		0.48 (0.21–1.05)	
21–25	22 (8.9)	50 (20.2)		0.45 (0.22–0.94)*	
26–30	29 (11.7)	40 (16.2)		0.77 (0.37–1.67)	
≥31	27 (10.9)	28 (11.3)		1	
Religion					
Orthodox	13 (5.3)	43 (17.4)		0.40 (0.20–0.81)*	
Protestant	5 (2.0)	8 (3.2)		0.83 (0.26–2.50)	
Muslim	76 (30.8)	102 (41.3)		1	
Ethnicity					
Others	11 (4.5)	29 (11.7)		0.48 (0.22–1.01)	
Amhara	18 (7.3)	44 (17.8)		0.50 (0.27–0.95)*	
Afar	62 (26.3)	80 (32.4)		1	
Residence					
Urban	58 (23.5)	129 (52.2)		0.30 (0.16–0.56)*	
Rural	36 (14.6)	24 (9.7)		1	
Educational status					
Illiterate	52 (21.1)	37 (15)		5.1 (2.64–9.87)*	4.20 (1.5–10.80)*
Primary school	23 (9.3)	47 (19.0)		1.78 (0.87–3.62)	1.51 (0.93–3.00)
Secondary school and above	19 (6.9)	69 (18.6)		1	1
Occupation					
Housewives	82 (33.2)	105 (42.5)		2.44 (1.09–5.56)*	
Government-employed	3 (1.2)	20 (8.1)		0.48 (0.11–2.00)	
Merchants	9 (3.6)	28 (11.3)		1	
Average monthly income (ETB)					
<1500	36 (14.6)	17 (6.9)		8.02 (3.52–18.28)*	5.62 (2.40–15.98)*
1500–3000	44 (17.8)	83 (33.6)		2.01 (1.00–4.01)	1.82 (0.95–3.76)
>3000	14 (5.6)	53 (21.4)		1	1
Family size					
≤4	54 (21.9)	125 (50.6)		0.30 (0.17–0.54)*	0.17 (0.08–0.71)*
>4	40 (16.2)	28 (11.3)		1	1
Birth interval					
<2 yr	35 (21.0)	20 (12.0)		2.94 (1.43–5.56)*	3.70 (1.15–12.50)*
≥2 yr	42 (25.1)	70 (41.9)		1	1
Parity					
Nulliparous	18 (7.3)	58 (23.5)		0.38 (0.21–0.71)*	
Multiparous	76 (30.8)	95 (38.5)		1	
Number of antenatal care utilization					
No prior antenatal care utilization	37 (15)		44 (17.8)	2.33 (1.04–5.00)*	5.88 (1.35–25.00)*
1–2 times visits	45 (18.2)		76 (30.8)	1.64 (0.76–3.33)	0.91 (0.28–3.33)
3–4 times visits	12 (4.9)		33 (13.4)	1	1
Frequency of eating per day					
3 times	85 (34.4)		98 (39.7)	8.33 (1.96–33.33)*	
4 times	7 (2.8)		35 (14.2)	2.00 (0.38–10.53)	
>4 times	2 (0.8)		20 (8.1)	1	
Dietary diversity					
Low	83 (33.6)		99 (40.1)	4.12 (1.96–8.65)*	3.45 (1.78–6.89)*
Adequate	11 (4.4)		54 (21.9)		
Food taboos					
Yes	54 (22)		39 (15.4)	4.00 (2.27–6.67)*	
No	40 (16.2)		114 (46.2)	1	

Others include Oromo, Southern Nations, Nationalities, and Peoples (SNNP), and Tigray ethnic groups.

AOR = adjusted odds ratio, CI = confidence interval, COR = crude odds ratio, ETB = Ethiopian birr, MUAC = mid-upper arm circumference.

* Statistically significant (*P* < .05).

### 3.2. Qualitative results

#### 3.2.1. Participant characteristics

Thirty-six pregnant women participated in 6 FGDs, with 4 FGDs conducted among urban residents and 2 among rural residents. Each FGD included 6 participants. Approximately 44.4% of participants were 21 to 30 years of age, 38.8% were older than 30 years, and 16.8% were younger than 20 years. Nearly one-third (30.6%) of participants were unable to read and write, while 47.2% had completed primary education. Most participants (88.9%) were multiparous. More than half (55.6%) were between 3 and 6 months of gestation, and approximately half had attended 1 ANC visit.

#### 3.2.2. Theme 1: food taboos during pregnancy

One overarching theme, food taboos during pregnancy, emerged from the analysis. Three interrelated subthemes were identified: avoidance of protein-rich foods because of fear of fetal overgrowth and difficult delivery; avoidance of semi-solid foods, fruits, and vegetables because of the belief that these foods adhere to the fetus; and avoidance of selected beverages because of perceived adverse fetal effects (Table 6).

**Table 6:** Framework thematic analysis of pregnancy-related food taboos among pregnant women.

Theme	Categories (subthemes)	Codes
Food taboos during pregnancy	Fear of fetal overgrowth and difficult delivery	Meat, eggs, milk, bread, Ashara (coffee with milk and sugar), “big fetus,” prolonged labor, difficult delivery
	Belief that foods adhere to the fetus	Porridge (Atmit), cereal soup, bananas, cabbage, yogurt, semi-solid foods, “stick to the fetus,” abnormal fetal development
	Fear of adverse fetal effects	Coca-Cola, Sprite, “thin fetal bones,” unhealthy fetal growth

##### 3.2.2.1. Subtheme 1: fear of fetal overgrowth and difficult childbirth

Most participants reported avoiding protein-rich foods, including meat, eggs, milk, and other animal products, because they believed these foods increased fetal size, resulting in prolonged labor and difficult delivery.

*“In our society, pregnant women do not eat meat, eggs, or milk because these foods make the fetus too big and delay delivery.”*

(Pregnant woman, FGD2)

Similarly, another participant explained:

*“I did not consume meat, eggs, or bread because they increased the baby’s weight, prolonged labor, and caused excessive bleeding.”*

(Pregnant woman, FGD4)

##### 3.2.2.2. Subtheme 2: belief that foods adhere to the fetus

Participants also believed that semi-solid foods, fruits, and vegetables could adhere to the fetus, resulting in abnormal fetal development or difficult childbirth. Foods commonly avoided included porridge, cereal soup (Atmit), bananas, cabbage, and yogurt.

*“Porridge, cereal soup (Atmit), bananas, and cabbage were avoided during pregnancy because they stick to the unborn baby.”*

(Pregnant woman, FGD1)

*“Yogurt, bananas, and porridge cling to the baby’s body, so I avoided eating them during pregnancy.”*

(Pregnant woman, FGD3)

##### 3.2.2.3. Subtheme 3: avoidance of beverages

Several participants reported avoiding coffee mixed with milk and sugar (locally known as Ashara) and soft drinks because of beliefs that these beverages either increased fetal weight or adversely affected fetal bone development.

*“In Afar culture, coffee mixed with milk and sugar (Ashara) is avoided because it increases the baby’s weight and makes delivery difficult.”*

(Pregnant woman, FGD5)

*“We avoided Coca-Cola, Sprite, porridge, and cereal soup because soft drinks weaken the baby’s bones and semi-solid foods stick to the fetus.”*

(Pregnant woman, FGD6)

### 3.3. Integration of quantitative and qualitative findings

The qualitative findings complemented the quantitative results by providing cultural explanations for the high prevalence of low dietary diversity (73.5%) observed among pregnant women. Although food taboo practice was not independently associated with maternal undernutrition after multivariable adjustment, the FGDs demonstrated that culturally driven avoidance of protein-rich foods, fruits, vegetables, and other nutrient-dense foods may contribute to poor dietary diversity. These findings suggest that food taboos may indirectly influence maternal nutrition through their effects on dietary practices rather than acting as an independent association with maternal undernutrition.

## 4. Discussion

To our knowledge, this is the first facility-based, mixed-methods study to investigate the prevalence of maternal undernutrition, its associated factors, and pregnancy-related food taboos among pregnant women attending ANC in the Afar Region, Ethiopia. Maternal undernutrition remains an important public health concern because it is associated with adverse maternal and neonatal outcomes, including intrauterine growth restriction, preterm birth, low birth weight, and increased perinatal morbidity and mortality.^[1–5]^ The mixed-methods approach enabled us to quantify the burden of undernutrition while also exploring the sociocultural beliefs that may influence maternal dietary practices.

The prevalence of maternal undernutrition in the present study was 38.1%, indicating that more than one-third of pregnant women were undernourished. This prevalence was lower than that reported in India^[8]^ but higher than the findings from previous studies conducted in eastern Ethiopia, Gondar, and Gambella, and higher than the national estimate reported in the 2016 Ethiopia Demographic and Health Survey.^[10–13]^ These differences may be explained by variations in study populations, study settings, measurement criteria for undernutrition (including differences in MUAC cutoff values), and contextual socioeconomic and environmental factors. The prevalence observed in the present study was comparable to the regional estimate reported for the Afar Region in the 2016 Ethiopia Demographic and Health Survey,^[10]^ suggesting that maternal undernutrition remains a persistent public health problem in the region. The high prevalence of maternal undernutrition observed in this study may reflect the cumulative effects of recurrent drought, food insecurity, the predominantly pastoral lifestyle, and limited access to diversified foods in the Afar Region.^[23]^ Although government and humanitarian nutrition programs have been implemented in the region, persistent environmental and socioeconomic challenges may continue to influence maternal nutritional status. In addition, cultural beliefs regarding food consumption during pregnancy may further contribute to inadequate dietary intake. However, these explanations should be interpreted cautiously because the present cross-sectional study was not designed to establish causal relationships.

Educational status was independently associated with maternal undernutrition. Pregnant women who were illiterate had higher odds of being undernourished than those who had attained secondary education or higher. This finding is consistent with previous studies conducted in Ethiopia and other low-income countries.^[8,15,16]^ Women with higher educational attainment may have greater access to nutrition information, better health literacy, and improved utilization of maternal healthcare services, which may contribute to healthier dietary practices and a reduced risk of undernutrition during pregnancy. Similarly, pregnant women from households with lower monthly incomes had higher odds of being undernourished than those from households with higher monthly incomes. This finding agrees with previous studies^[8,15,16]^ and may reflect reduced household purchasing power and limited access to diverse and nutrient-rich foods among economically disadvantaged families. Limited financial resources may constrain the ability of households to meet the increased nutritional requirements of women during pregnancy. Household family size was another important factor associated with maternal undernutrition. Women living in households with 4 or fewer family members had significantly lower odds of being undernourished than those living in households with more than 4 family members. This finding is consistent with previous studies.^[17,18]^ Larger household size may increase competition for available household food resources, potentially reducing the quantity and quality of food available to pregnant women and consequently increasing their risk of undernutrition. A birth interval of less than 2 years was independently associated with higher odds of maternal undernutrition. This finding is consistent with previous studies^[17,18]^ and may be explained by inadequate maternal nutritional recovery between successive pregnancies, which can result in depletion of maternal nutrient stores and increased nutritional demands during subsequent pregnancies. ANC utilization was also independently associated with maternal undernutrition. Pregnant women with no prior ANC utilization during the current pregnancy had significantly higher odds of being undernourished than those with 3 to 4 prior ANC visits. Similar findings have been reported in previous Ethiopian studies.^[20,21]^ ANC provides opportunities for nutrition counseling, iron and folic acid supplementation, early identification of nutritional problems, and reinforcement of appropriate dietary practices during pregnancy, which may contribute to improved maternal nutritional status. Low dietary diversity was also significantly associated with maternal undernutrition. Pregnant women with low dietary diversity had higher odds of being undernourished than those with adequate dietary diversity. This finding is consistent with evidence from southern Ethiopia^[19]^ and highlights the importance of consuming a variety of food groups to meet the increased nutritional requirements during pregnancy. Inadequate dietary diversity may indicate insufficient intake of essential nutrients and may therefore contribute to maternal undernutrition.

Unlike previous findings from southern Ethiopia,^[19]^ meal frequency was not independently associated with maternal undernutrition after adjustment for potential confounding variables. This discrepancy may reflect differences in dietary quality, study populations, and local feeding practices rather than meal frequency alone. Similarly, although food taboo practice was significantly associated with maternal undernutrition in the bivariable analysis, the association was no longer statistically significant after adjustment for potential confounding variables. This suggests that the observed crude association may have been explained by other socioeconomic, demographic, or maternal factors included in the multivariable model. This finding differs from a study conducted in Nigeria.^[14]^ The lack of an independent association may indicate that food taboos influence maternal undernutrition indirectly through reduced dietary diversity and other socioeconomic factors rather than being independently associated with maternal nutritional status.

The qualitative findings complemented the quantitative results by providing important contextual explanations for the high prevalence of low dietary diversity observed among pregnant women. Participants consistently reported avoiding protein-rich foods (meat, eggs, and milk), semi-solid foods, fruits, vegetables, and selected beverages because of cultural beliefs that these foods increase fetal size, complicate delivery, adhere to the fetus, or adversely affect fetal development. Although food taboo practices were not independently associated with undernutrition after multivariable adjustment, these qualitative findings suggest that culturally driven food restrictions may reduce dietary diversity and contribute to inadequate maternal nutrient intake. The integration of quantitative and qualitative findings therefore provides a more comprehensive understanding of maternal undernutrition in this pastoral community.

### 4.1. Strengths and limitations of the study

A major strength of this study is the use of a mixed-methods approach, which enabled triangulation of quantitative and qualitative findings and provided a more comprehensive understanding of maternal undernutrition and culturally related dietary practices among pregnant women attending ANC in the Afar Region.

Nevertheless, some limitations should be acknowledged. First, the cross-sectional design precludes establishing temporal or causal relationships between the identified factors and maternal undernutrition. Second, because the study was facility-based and included only women attending ANC services, the findings may not be generalizable to all pregnant women in the Afar Region, particularly those who do not utilize health services. Third, self-reported information on dietary practices and food taboos may have been affected by recall bias and social desirability bias. Fourth, detailed quantitative dietary intake assessments and biochemical indicators of nutritional status, including hemoglobin concentration and serum micronutrient measurements, were not collected. Additionally, some regression categories contained relatively small numbers of observations, which may have reduced the precision of some adjusted estimates, as reflected by the wide CIs. Finally, although the qualitative component strengthened interpretation of the findings, its transferability may be limited to similar pastoral settings. Future longitudinal community-based studies incorporating comprehensive dietary assessments, physical activity, micronutrient biomarkers, and broader geographic representation are warranted.

## 5. Conclusion

Maternal undernutrition remains a substantial public health problem among pregnant women attending ANC at Awash Sebat Health Center in the Afar Region, with more than one-third of women classified as undernourished. Maternal education, household income, family size, birth interval, ANC utilization, and dietary diversity were independently associated with maternal undernutrition. The qualitative findings suggested that culturally rooted food taboos may contribute to the avoidance of several nutrient-rich foods during pregnancy and may indirectly reduce dietary diversity. Therefore, interventions to reduce maternal undernutrition should strengthen female education, promote household economic empowerment, encourage adequate birth spacing through family planning, improve ANC utilization, and provide culturally sensitive nutrition education that addresses misconceptions about food taboos while respecting local beliefs and practices.

## Acknowledgments

The authors sincerely thank all study participants, data collectors, supervisors, and the staff of Awash Sebat Health Center for their valuable cooperation during the study. The authors also acknowledge Samara University for providing ethical approval and administrative support.

## Author contributions

**Conceptualization:** Temesgen Gebeyehu Wondmeneh, Molla Kahssay.

**Data curation:** Temesgen Gebeyehu Wondmeneh, Molla Kahssay.

**Formal analysis:** Temesgen Gebeyehu Wondmeneh, Molla Kahssay.

**Funding acquisition:** Temesgen Gebeyehu Wondmeneh, Molla Kahssay.

**Investigation:** Temesgen Gebeyehu Wondmeneh, Molla Kahssay.

**Methodology:** Temesgen Gebeyehu Wondmeneh, Molla Kahssay.

**Project administration:** Temesgen Gebeyehu Wondmeneh, Molla Kahssay.

**Resources:** Temesgen Gebeyehu Wondmeneh, Molla Kahssay.

**Software:** Temesgen Gebeyehu Wondmeneh, Molla Kahssay.

**Supervision:** Temesgen Gebeyehu Wondmeneh, Molla Kahssay.

**Validation:** Temesgen Gebeyehu Wondmeneh, Molla Kahssay.

**Visualization:** Temesgen Gebeyehu Wondmeneh, Molla Kahssay.

**Writing – original draft:** Temesgen Gebeyehu Wondmeneh, Molla Kahssay.

**Writing – review & editing:** Temesgen Gebeyehu Wondmeneh, Molla Kahssay.
